# Parallel coding of first and second order stimulus attributes

**DOI:** 10.1186/1471-2202-13-S1-O13

**Published:** 2012-07-16

**Authors:** Patrick McGillivray, Katrin Vonderschen, Eric S Fortune, Maurice J Chacron

**Affiliations:** 1 Department of Physiology, McGill University, Montreal, QC, Canada; 2 Department of Psychological and Brain Sciences, Johns Hopkins University, Baltimore, MD, USA; 3 Department of Neuroscience, Johns Hopkins University, Baltimore, MD, USA; 4 Museum of Zoology, Pontificia Universidad Católica del Ecuador, Quito, Ecuador; 5 Department of Physics, McGill University, Montreal, QC, Canada

## 

Natural stimuli often have time varying first (i.e. mean) and second order (i.e. variance) attributes that each carry critical information for perception and can vary independently over orders of magnitude. We recorded the responses of midbrain electrosensory neurons in the weakly electric fish *Apteronotus leptorhynchus* to stimuli with first and second order attributes that varied independently in time. We found two distinct groups of midbrain neurons: the first group responded to both first and second order attributes while the other responded selectively to second order attributes. Using computational analyses, we show how inputs from a heterogeneous population of ON- and OFF-type afferent neurons are combined in order to give rise to response selectivity to second order stimulus attributes in midbrain neurons. Our study thus uncovers, for the first time, generic and widely applicable mechanisms by which selectivity to second order stimulus attributes emerges in the brain.

